# Recent Advances in Fucoidan Bioactivity and Its Molecular Mechanisms in the Intervention of Vascular Cognitive Impairment

**DOI:** 10.3390/md24100341

**Published:** 2026-09-28

**Authors:** Jianing Wang, Yingrui Qu, Meng Sun, Yumeng Zhao, Yihang Bai, Jingxuan Shi, Yibo Li, Chao Gao, Ming Yu, Wenwei Han

**Affiliations:** 1 Shandong Key Laboratory of Neurorehabilitation, Qingdao Hospital, University of Health and Rehabilitation Sciences (Qingdao Municipal Hospital), Qingdao 266071, China; 2025022064@qdu.edu.cn (J.W.); quyingrui@uhrs.edu.cn (Y.Q.); sunm2208@163.com (M.S.); zhaoym31415@outlook.com (Y.Z.); a449177b@gmail.com (Y.B.); 13220511235@163.com (J.S.); 17353366876@163.com (Y.L.); gcicxy@163.com (C.G.); 2 School of Pharmacy, Qingdao University Medical College, Qingdao 266073, China; 3 NHC Key Laboratory of Cardiopulmonary Rehabilitation and Functional Recovery, University of Health and Rehabilitation Sciences, Qingdao 266071, China

**Keywords:** fucoidan, low-molecular-weight fucoidan, vascular cognitive impairment, neuroinflammation, biological activity, molecular mechanisms

## Abstract

Fucoidan is a class of sulfated polysaccharides primarily derived from marine organisms, particularly brown algae and some marine invertebrates. Owing to its structural diversity and broad spectrum of biological activities, including immunomodulatory, anti-inflammatory, antioxidant, vascular protection, anticoagulant, and neuroprotective effects, fucoidan has attracted increasing attention as a promising candidate for functional foods and pharmaceutical applications. This review systematically summarizes the sources and distribution of fucoidan, modern extraction and preparation strategies, chemical structural characteristics, and structure–activity relationships. The biological activities and potential mechanisms of action of fucoidan were the primary focus of this study. Furthermore, this review provides an overview of the molecular mechanisms underlying the protective effects of fucoidan against vascular cognitive impairment. These mechanisms mainly involve the inhibition of NF-κB- and MAPK-mediated neuroinflammatory responses, activation of the Nrf2/HO-1 antioxidant pathway to alleviate oxidative stress, regulation of microglial polarization, maintenance of blood–brain barrier integrity, promotion of neurotrophic factor expression, modulation of autophagy and apoptosis-related pathways, and attenuation of microvascular damage induced by chronic cerebral hypoperfusion through VEGF signaling. Given its multi-target and multi-pathway regulatory properties, fucoidan exhibits promising potential for the prevention and management of vascular cognitive impairment and related neurodegenerative disorders.

## 1. Introduction

Fucoidan is a sulfated natural polysaccharide primarily derived from brown algae, including *Laminaria*, *Undaria*, and *Ecklonia* species, as well as certain marine invertebrates. Its unique structural characteristics, including abundant L-fucose residues and sulfate groups, contribute to its diverse biological activities [1]. Numerous studies have demonstrated that fucoidan exhibits a broad range of bioactivities, including immunomodulatory, anti-inflammatory, antioxidant, antitumor, antiviral, and gut microbiota-regulating effects. Owing to these multifunctional properties, fucoidan has attracted considerable attention as a promising bioactive compound for applications in biomedicine, functional foods, and cosmeceuticals [2].

With the global population aging, vascular cognitive impairment (VCI) has emerged as a significant public health concern [3]. VCI is primarily associated with cerebrovascular abnormalities, including cerebral ischemia, cerebral hemorrhage, and chronic cerebral hypoperfusion. Clinically, VCI is characterized by progressive cognitive decline, including memory impairment and executive dysfunction, and frequently exhibits pathological interactions or comorbidity with neurodegenerative diseases such as Alzheimer’s disease. The underlying pathological mechanisms of VCI are highly complex and involve multiple factors, including oxidative stress, neuroinflammation, blood–brain barrier (BBB) disruption, and apoptosis-related signaling pathways [4]. Currently, effective and safe therapeutic strategies capable of delaying or reversing VCI progression remain unavailable, and clinical treatment options are still severely limited.

In recent years, the neuroprotective potential of fucoidan has attracted increasing attention, particularly regarding its application in the prevention and management of VCI [5]. The diverse biological activities of fucoidan closely correspond to the key pathological mechanisms underlying VCI, highlighting its potential for multi-target and multi-pathway regulation [6]. This review provides a comprehensive overview of the natural sources and resource distribution of fucoidan, as well as recent advances in its extraction, purification, and structural modification. Furthermore, the chemical structure of fucoidan and the structure–activity relationships underlying its biological effects are systematically summarized. In addition, the major biological activities of fucoidan, including antioxidant, anti-inflammatory, immunomodulatory, and anti-apoptotic effects, together with their underlying molecular mechanisms, are discussed. This review particularly focuses on recent advances in the protective effects of fucoidan against VCI and summarizes its mechanisms of action at the molecular and cellular levels. These mechanisms include attenuation of oxidative stress, suppression of neuroinflammation, regulation of microglial polarization, preservation of BBB integrity, enhancement of neurotrophic factor expression, and modulation of autophagy- and apoptosis-related pathways. Finally, the potential applications of fucoidan in preventing and ameliorating cognitive decline are discussed, along with future research directions from the perspectives of molecular mechanisms and translational applications. However, several challenges remain in the clinical translation of fucoidan, including the need for systematic elucidation of structure–activity relationships and multi-target regulatory networks. Addressing these challenges will facilitate the development of fucoidan-based functional foods and neuroprotective products and provide valuable insights for future research and applications.

To enhance the transparency and reproducibility of this review, a narrative literature search was conducted to identify studies on fucoidan and vascular cognitive impairment (VCI) using PubMed, Web of Science, and China National Knowledge Infrastructure (CNKI), with the search conducted up to September 2026. The search terms included “fucoidan,” “vascular cognitive impairment,” “vascular dementia,” “chronic cerebral hypoperfusion,” “neuroinflammation,” “oxidative stress,” “blood–brain barrier,” “angiogenesis,” “coagulation,” and “gut–brain axis,” as well as their related terms. Studies directly addressing VCI or its associated pathological processes were prioritized. Studies using other diseases or experimental models were also considered when direct evidence in VCI was limited, provided that they offered relevant mechanistic insights. These studies were included as indirect evidence or as supportive evidence for proposed mechanisms.

## 2. Structural Characteristics and Preparation Methods

### 2.1. Sources and Distribution Characteristics of Fucoidan

Fucoidan is a class of sulfated polysaccharides derived from marine organisms, such as brown algae, sea cucumbers and sea urchins, and is widely distributed across various natural sources. Previous studies have demonstrated that fucoidan can be extracted from a variety of brown algae, including the genera *Sargassum*, *Laminaria*, and *Undaria*, as well as kelp species [7,8,9,10]. These brown algae are widely distributed in oceans around the world, providing abundant raw materials for the large-scale production of fucoidan. It should be noted that species, harvesting location, season, seawater temperature, and growth conditions can significantly influence the extraction yield, monosaccharide composition, degree of sulfation, and molecular weight distribution of fucoidan [11]. A study of kelp collected at different latitudes has shown that the molar ratio of fucose to sulfate in its extracts fluctuates significantly with geographical latitude. These compositional differences may directly affect the immunomodulatory activities of fucoidan extracts, particularly their regulatory effects on immune cells such as natural killer (NK) cells [12]. Therefore, these critical factors should be carefully considered during the research, development, and application of fucoidan-based products.

### 2.2. Chemical Structure and Structure-Activity Relationships of Fucoidan

Fucoidan generally exhibits a highly complex and heterogeneous structure and belongs to a class of sulfated branched polysaccharides. Its biological activities are strongly dependent on its physicochemical properties, degree and distribution pattern of sulfation, sulfation positions, and spatial branching architecture [13,14].

The structure of fucoidan varies considerably depending on the source species of seaweed. Structurally, fucoidan can be broadly classified into two major structural types. Type I fucoidan is primarily composed of repeating α-(1 → 3)-linked L-fucose units, forming a relatively regular linear backbone. Sulfation predominantly occurs at the hydroxyl groups located at the C-2 and C-4 positions of L-fucose residues, whereas the C-3 position is generally unsubstituted by sulfate groups. In contrast, Type II fucoidan consists of alternating α-(1 → 3)- and α-(1 → 4)-linked L-fucose units, resulting in a more flexible backbone with distinct conformational properties. Sulfation commonly occurs at the C-2 and/or C-4 positions, with a smaller proportion of sulfate groups located at the C-3 position, leading to greater diversity in substitution patterns [15,16] (Figure 1).

It should be noted that most fucoidans isolated from different algal species are heteropolysaccharides. The precise distribution and linkage patterns of other monosaccharide components, including mannose, galactose, glucose, xylose, rhamnose, and arabinose, remain incompletely characterized. These structural variations contribute to the remarkable complexity and diversity of fucoidan molecules. Fucoidans from different sources exhibit significant differences in structural parameters such as sugar composition, sulfate content, molecular weight, and glycosidic-linkage patterns. The correspondence between their structural characteristics and the reported biological activities is summarized in Supplementary Materials Table S1 [9,10,17,18,19,20,21,22,23,24].

#### 2.2.1. Molecular Weight

Molecular weight is one of the important structural parameters influencing the biological functions of fucoidan. Naturally extracted fucoidan preparations often exhibit a broad molecular weight distribution, ranging from several kilodaltons to several hundred kilodaltons. In general, high-molecular-weight fucoidan (HMWF; typically, >30 kDa) exhibits higher viscosity and greater structural complexity and may possess stronger anticoagulant activity. HMWF has shown potential advantages in immune activation in vitro [25], intestinal mucosal barrier repair, lipid metabolism regulation, and prebiotic effects. These effects are thought to be mediated, at least in part, through modulation of the gut microbiota and immune cells located at the intestinal mucosal interface, thereby contributing to systemic physiological regulation [26]. In contrast, low-molecular-weight fucoidan (LMWF; typically, <30 kDa) generally exhibits better solubility, lower viscosity, and potentially greater tissue accessibility. In specific disease models, LMWF maintains strong antioxidant activity and may interact more efficiently with cell surface receptors, such as scavenger receptors and Toll-like receptors, thereby exhibiting enhanced regulatory effects on specific molecular targets [10,19]. However, molecular weight is not the sole determinant of fucoidan activity. The degree of sulfation, the position of sulfate groups, the degree of branching, and monosaccharide composition can also significantly influence its interactions with proteins, receptors, and growth factors (see Supplementary Materials Table S2 for details [27,28,29,30,31,32,33,34,35,36,37,38,39,40]).

#### 2.2.2. Sulfate Content

As a key functional group of fucoidan, sulfate groups are commonly substituted at the C-2 or C-4 positions of L-fucopyranosyl residues, whereas substitution at the C-3 position exhibits a more variable distribution pattern [41]. The sulfation pattern and substitution sites of sulfate groups are important determinants of the biological activities of sulfated polysaccharides. In low-molecular-weight fucoidan, increasing sulfate content from 18.7% to 32.1% gradually reduces anti-lipogenic activity while significantly enhancing anti-inflammatory and antioxidant activities [36].

Accumulating evidence suggests that the anticoagulant activity of fucoidan is closely associated with its sulfate content, sulfation pattern, molecular weight, and monosaccharide composition. Moderate sulfation, particularly sulfation at the C-2 position and disulfation at the C-2 and C-3 positions, has been suggested to favor anticoagulant activity. In contrast, excessive sulfation may reduce both anticoagulant and antithrombin activities [42,43].

#### 2.2.3. Branched Chain

The branched structure of fucoidan represents one of the major factors contributing to its high structural heterogeneity. Although the main chain consists primarily of α-L-fucose residues, the side chains are commonly composed of monosaccharides such as galactose, mannose, glucose, glucuronic acid, and small amounts of xylose and rhamnose. These side chains can be attached to the C-2, C-3, or C-4 position of fucose residues, resulting in highly complex branched architectures [44]. Fucoidan preparations obtained from different sources and extraction processes can differ substantially in the number and length of branches, monosaccharide composition, and sulfation sites, providing a structural basis for the diversity of their biological activities [45]. Oliveira et al. [46] reported that both the position of sulfate groups and the degree of branching can significantly affect the biological activities of fucoidan, including its antioxidant, anti-inflammatory, and anticoagulant properties. At present, evidence regarding the structure–activity relationships of fucoidan is derived predominantly from non-VCI models, including those of cancer, metabolic disorders, and inflammatory diseases. Direct evidence in the context of VCI or chronic cerebral hypoperfusion (CCH) remains limited. Therefore, the effects of molecular weight, degree of sulfation, branching pattern, and monosaccharide composition on the neuroprotective effects of fucoidan in VCI are currently inferred largely from indirect evidence. Given the pathological features of VCI, including chronic hypoperfusion, oxidative stress, neuroinflammation, and blood–brain barrier dysfunction, fucoidan fractions with anti-inflammatory, antioxidant, and endothelial-protective properties may have greater therapeutic potential. In addition, angiogenesis may exert either protective or pathological effects depending on disease stage. However, its effects on VEGF-associated angiogenesis appear to be highly context-dependent, indicating that the structure–activity relationships of fucoidan in VCI require further validation in relevant models, such as CCH models.

### 2.3. Modern Techniques for the Preparation of Fucoidan

Fucoidan is typically obtained through mild extraction processes directly from brown algae (such as kelp and wakame), with the primary objective of preserving the integrity of the macromolecular chains and minimizing structural degradation. The crude extracts are subsequently purified by removing impurities, such as proteins, pigments and other non-polysaccharide components, thereby yielding relatively homogeneous fucoidan preparations. To address the issues of high molecular weight and poor solubility associated with natural fucoidan, physical, chemical, or biological approaches have been employed to reduce its molecular weight, and enhance its potential and bioactivity. In recent years, modern green and environmentally friendly extraction technologies have been rapidly developed to maximize the preservation of fucoidan’s native structure and biological properties [47].

#### 2.3.1. Traditional Methods

Traditional fucoidan extraction methods mainly involve hot water extraction and acid–alkali extraction. These methods are often associated with long extraction times and relatively low yields. Moreover, the use of strong acids and alkalis can easily damage the polysaccharide backbone and cause the loss of sulfate groups, thereby affecting the structural integrity and biological activities of fucoidan [7]. Modern technologies are primarily focused on improving efficiency while preserving the native structure and functional properties of fucoidan. Physically assisted extraction technologies, such as microwave-assisted extraction (MAE) and ultrasound-assisted extraction (UAE), are widely used due to their high efficiency and speed. The cavitation effect of ultrasound can disrupt the cell walls of seaweed, accelerating solvent penetration. Previous studies have shown that 35 kHz ultrasonic treatment or microwave technology can significantly improve the extraction yield of fucoidan from brown algae. The resulting extract exhibits enhanced in vitro free radical scavenging activities [48,49]. By applying response surface methodology to systematically optimize the extraction system’s temperature, time, liquid-to-solid ratio and ultrasonic amplitude, the researchers successfully maximized both fucoidan yield and bioactivity [50,51]. In addition, ultrasound technology is also used for the secondary recovery of by-products (such as alginates) following the extraction of fucoidan, thereby contributing to the comprehensive and sustainable utilization of marine biomass resources [52].

#### 2.3.2. Biosynthetic Technology

In the field of biosynthesis, strategies integrating engineered microbial strains with enzymatic catalysis enable the precise production of high-purity and structurally defined fucose derivatives. These approaches provide potential solutions to the challenges associated with the high structural heterogeneity of natural fucoidan and the irregular distribution of sulfation sites within its polysaccharide chains [53]. Research has shown that GDP-L-fucose serves as a key precursor for fucose glycosylation and biosynthesis, and its stable intracellular supply is critical for improving biosynthetic efficiency. By inducing overexpression of the fkp gene in yeast to reconstruct the fucose biosynthetic pathway, combined with genomic integration and metabolic flux regulation, researchers have successfully achieved efficient and stable synthesis of free L-fucose [54]. Enzymatic conversion of GDP-L-fucose provides a more direct approach for L-fucose biosynthesis, thereby simplifying the production process and reducing costs [55]. Beyond chemical synthesis approaches, bioenzymatic catalysis can also facilitate the precise modification and preparation of fucoidan derivatives. Using GDP-fucose as a donor substrate and employing engineered enzymes, such as fucosyltransferases and sulfotransferases, glycosidic bond formation and sulfation modification can be catalyzed in a controlled manner. Silchenko et al. [56] utilized the endo-fucoidanase FWf5 to selectively prepare two fucoidan derivatives with distinct sulfation patterns. Among these derivatives, the polymeric 2,4-di-O-sulfated derivative exhibited higher in vitro anticoagulant activity. This study revealed previously unrecognized substrate specificity within the GH168 family of fucoidanases and provides new opportunities for producing standardized fucoidan derivatives with enhanced anticoagulant activity. Furthermore, fucoidan biosynthesis in brown algae involves the participation of eight genes [57]. Following the conversion of fructose-6-phosphate into GDP-mannose by three upstream enzymes, fucoidan biosynthesis proceeds through the coordinated actions of key enzymes involved in two pathways: de novo synthesis and the salvage pathway [57]. To date, this biosynthetic pathway has been confirmed only in kelp species and bacteria [58].

#### 2.3.3. Supercritical Extraction

Supercritical fluid extraction (SCFE) is a promising green extraction technology that utilizes fluids in a supercritical state, typically carbon dioxide (CO_2_), as extraction solvents. Owing to their gas-like diffusivity and liquid-like solvating capacity, supercritical fluids enable the efficient extraction of a wide range of bioactive compounds [59]. Previous studies have optimized the process conditions for supercritical CO_2_ extraction of spirulina and demonstrated that the resulting crude polysaccharide extracts contained 9.25% monosaccharides, including five monosaccharide components such as rhamnose and xylose [60]. As the most commonly used supercritical solvent, CO_2_ exhibits a low critical temperature and pressure, tunable solvent properties, and excellent mass transfer characteristics. Furthermore, because CO_2_ exists as a gas under standard temperature and pressure conditions, it facilitates solvent recovery and enables residue-free analysis [61]. SCFE also has the potential to preserve the native structure of fucoidan, enrich highly purified and structurally consistent active components, and improve product stability [62,63]. Relevant studies have shown that pretreatment of brown algae with supercritical CO_2_ (550 bar, 60 °C) produced fucoidan yields and structural characteristics comparable to those obtained using conventional ethanol-based extraction methods. However, when 5% ethanol was combined with supercritical CO_2_, low-sulfated fractions and heterogeneous monosaccharide components were effectively removed, resulting in fucoidan fractions enriched in highly sulfated components with more homogeneous monosaccharide compositions [64].

#### 2.3.4. Degradation Techniques for the Preparation of Low-Molecular-Weight Fucoidan

To address the relatively high molecular weight and limited solubility of naturally occurring fucoidan, physical, chemical, or biological degradation approaches can be employed to reduce its molecular weight and thereby improve its solubility and potential bioavailability. In recent years, considerable attention has been devoted to modern green extraction and degradation technologies, with the aim of preserving the native structural features and biological activities of fucoidan to the greatest extent possible [47]. Various approaches have been developed for the preparation of low-molecular-weight fucoidan (LMWF), each with distinct advantages and characteristics [65]. Physical methods generally provide better structural preservation. These methods include γ-ray irradiation [27,28], which can preserve sulfate groups and antioxidant activity; photocatalytic degradation [33], which is an efficient and environmentally friendly approach; and ultrasound-assisted degradation [35], whose depolymerization efficiency can be controlled by multiple processing parameters. Chemical methods are relatively simple but may cause undesirable side reactions. Common approaches include acid hydrolysis [30], which can cause desulfation; hydrogen peroxide-mediated degradation [36,37], which cleaves glycosidic bonds through reactive oxygen species; and hydrothermal degradation [39], which can reduce molecular weight while retaining sulfate groups. Enzymatic degradation uses specific endo-acting enzymes to hydrolyze α-L-fucoidan linkages. This approach offers good control over product structure and allows tailored preparation. However, its relatively high cost may limit large-scale applications [31].

## 3. Molecular Mechanisms of Fucoidan Bioactivity

Fucoidan exhibits diverse biological activities by interacting with various cell surface receptors and signaling molecules through its sulfate groups, thereby regulating downstream signaling pathways. These pathways are interconnected and act synergistically in diverse biological processes, including anti-inflammatory, antioxidant, anti-tumor, and immunomodulatory activities and maintenance of gut homeostasis, collectively forming a multilayered, multitarget regulatory network of biological functions (Figure 2).

### 3.1. Immune Regulatory Mechanisms

Fucoidan exerts anti-inflammatory effects by modulating the NF-κB and MAPK signaling pathways and inhibiting NF-κB nuclear translocation. In addition, it alleviates oxidative stress and reduces the expression of pro-inflammatory mediators, including COX-2, iNOS, TNF-α, and IL-6, thereby mitigating inflammatory responses [66].

#### 3.1.1. NF-κB Pathway

Sanjeewa et al. reported that, in zebrafish models, fucoidan suppresses the expression of TLR2 and TLR4 receptors, reduces downstream IKK-α activation, inhibits IκB-α phosphorylation, and enhances IκB-α stability. This process prevents the nuclear translocation of NF-κB (p50 and p65), thereby downregulating LPS-induced expression of iNOS, COX-2, and pro-inflammatory cytokines, including TNF-α, IL-1β, and IL-6. Consequently, NO production and cellular cytotoxicity are attenuated. These findings further demonstrate the anti-inflammatory potential of brown algae-derived fucoidan [67,68].

#### 3.1.2. MAPK Pathway

The MAPK signaling pathway mainly consists of three core branches: extracellular signal-regulated kinases 1 and 2 (ERK1/2), c-Jun N-terminal kinase (JNK), and p38 MAPK [69]. Inhibition of MAPK phosphorylation represents a potential therapeutic strategy for inflammation-related diseases [70]. Several studies have demonstrated that polysaccharides derived from brown algae can significantly reduce MAPK pathway phosphorylation levels [71]. Accumulating evidence indicates that fucoidans extracted from *Sargassum horneri* and *Sargassum autumnale* (SA) alleviate LPS-induced inflammatory responses in macrophages. These fucoidans exert anti-inflammatory effects by inhibiting ERK and p38 MAPK phosphorylation, thereby downregulating the expression of iNOS, COX-2, and pro-inflammatory cytokines, including TNF-α, IL-1β, and IL-6 [72,73]. Furthermore, the three downstream branches of the MAPK pathway interact with the NF-κB signaling pathway, collectively amplifying inflammatory cascades. Therefore, inhibition of MAPK and NF-κB activation may effectively attenuate excessive inflammatory responses [72,73].

#### 3.1.3. Scavenger Receptor A

The class A scavenger receptor (SR-A) is one of the key recognition receptors involved in fucoidan-mediated immune activation. SR-A can mediate intracellular signaling cascades; however, because its cytoplasmic N-terminus lacks a classical signaling sequence, the precise mechanism by which fucoidan initiates signaling pathways through SR-A remains unclear. It is widely proposed that this process requires coordination with membrane receptors, such as Toll-like receptors (TLRs) [74]. Previous studies have confirmed that SR-A is an important mediator of lipopolysaccharide (LPS)-induced inflammatory responses. This effect is primarily mediated through the TLR4-dependent NF-κB activation pathway [75]. Fucoidan suppresses endoplasmic reticulum stress (ERS)-mediated autophagy in macrophages by activating the AKT/mTOR/p70S6K pathway through SR-A, thereby inducing macrophage apoptosis. Furthermore, this SR-A-mediated apoptotic process requires the synergistic involvement of TLR4 [74,76]. Negatively charged fucoidan acts as a ligand for SR-A. Following binding to SR-A on the macrophage surface, fucoidan activates downstream p38 and JNK signaling pathways, thereby contributing to biological processes including macrophage activation and inflammatory regulation [77].

#### 3.1.4. Toll-like Receptors

Toll-like receptors (TLRs) are a class of highly evolutionarily conserved transmembrane proteins that recognize both endogenous and exogenous ligands. Fucoidan activates IκB kinase (IKK) through TLR2/4-mediated signaling. This activation promotes NF-κB signaling and facilitates the rapid translocation of the NF-κB complex from the cytoplasm to the nucleus. Once translocated into the nucleus, NF-κB regulates the transcription of target genes, promotes the production of pro-inflammatory cytokines, and consequently modulates immune responses [78,79]. Teruya et al. demonstrated that fucoidan is recognized by TLR4, which interacts with scavenger receptor A (SR-A) and CD14, thereby activating the MAPK signaling pathway and inducing nitric oxide (NO) production in macrophages [80]. Fucoidan derived from Fucus vesiculosus inhibits lipopolysaccharide (LPS)-induced inflammatory responses in macrophages by suppressing the TLR4/NF-κB signaling pathway, thereby contributing to the regulation of the inflammatory microenvironment during immune activation [68,81].

#### 3.1.5. Mechanisms of the Immune Response to Viruses

The antiviral mechanisms of fucoidan can be broadly categorized into two aspects: inhibition of viral activity and enhancement of host antiviral immune responses. On the one hand, fucoidan binds to viral spike proteins (S proteins) through its sulfate groups, thereby blocking the interaction between viral particles and the angiotensin-converting enzyme 2 (ACE2) receptor and preventing viral entry [82]. On the other hand, previous findings from our laboratory indicate that fucoidan activates the STING–TBK1–IRF3 signaling pathway through STING, inducing the production of type I interferons (IFNs) and interferon-stimulated genes (ISGs), including IFIT1, IFIT2, and CXCL10. This process enhances innate antiviral immune responses [83].

Furthermore, C-type lectins (CLECs) and C-type lectin receptors (CLRs) not only recognize diverse antigens but also mediate intracellular signaling, thereby facilitating macrophages and dendritic cells in initiating innate immune responses [84].

Overall, the NF-κB/MAPK signaling axis can be considered a high-confidence candidate mechanism underlying the neuroprotective effects of fucoidan on VCI. Its potential importance lies in its involvement in multiple interconnected pathological processes, including inflammatory amplification, glial cell activation, BBB dysfunction, and neuronal apoptosis. Modulation of this pathway may therefore contribute to the multi-target therapeutic effects of fucoidan by interrupting the pathological cascade that drives VCI progression.

### 3.2. Antioxidant Mechanisms

The Keap1–Nrf2 signaling pathway is a key component of cellular defense against oxidative stress. Low-molecular-weight fucoidan isolated from the seaweed *Sargassum siliquastrum* activates the Nrf2 pathway, promoting Nrf2 nuclear translocation and upregulating the expression of antioxidant enzymes, including heme oxygenase-1 (HO-1) and NAD(P)H quinone oxidoreductase 1 (NQO1), as well as nuclear Nrf2 protein levels. These effects reduce Reactive Oxygen Species (ROS) and NO production and enhance antioxidant capacity by regulating cellular antioxidant systems [85]. Other studies have demonstrated that fucoidan isolated from fermented *Sargassum* alleviates oxidative stress by activating the Nrf2 signaling pathway, increasing catalase and superoxide dismutase (SOD) levels, and promoting intracellular ROS scavenging, thereby reducing H_2_O_2_-induced Vero cell death [86]. In a D-galactose-induced cognitive decline model, fucoidan activated the upstream APN/AMPK/SIRT1 signaling pathway, which subsequently stimulated the downstream PGC-1α/NRF1/TFAM pathway to promote mitochondrial biogenesis. This process improved mitochondrial dysfunction by alleviating oxidative stress and inflammation, ultimately ameliorating D-galactose-induced cognitive impairment [87]. Furthermore, a rotundine-induced rat model of Parkinson’s disease demonstrated that fucoidan specifically regulates the PGC-1α/Nrf2 signaling pathway, thereby maintaining mitochondrial homeostasis, reducing oxidative stress-induced damage, protecting the dopaminergic system, and improving motor dysfunction in model rats [88].

### 3.3. Anti-Tumor and Pro-Apoptotic Mechanisms

Additional mechanistic evidence from non-VCI disease models, particularly cancer studies, indicates that fucoidan regulates several signaling pathways relevant to VCI pathogenesis, including JAK/STAT3 [22], PI3K/AKT/mTOR [89,90,91,92,93,94,95,96], HIF-1α/VEGF [97,98], and ROS-mediated mitochondrial apoptosis [99]. Although these pathways have primarily been investigated in oncology, many are also involved in neuroinflammation, oxidative stress, endothelial dysfunction, angiogenesis, and neuronal survival during VCI progression. They therefore provide complementary evidence of the broad biological activities of fucoidan rather than direct evidence for its effects on VCI. Accordingly, this is briefly discussed in the present review to highlight the multitarget and multi-pathway characteristics of fucoidan, rather than serving as a major focus of the VCI discussion.

### 3.4. Procoagulant/Anticoagulant Mechanisms

Fucoidan, a class of sulfated polysaccharides, exhibits both procoagulant and anticoagulant activities through the regulation of platelet function. The interaction between fucoidan and platelet membrane glycoprotein GPIbα plays a critical role in this process. Fucoidan binds to the extracellular domain of GPIbα and interferes with the interaction between GPIbα and von Willebrand factor (vWF). However, fucoidan can also initiate GPIbα-dependent signaling, which subsequently activates integrin αIIbβ3 and promotes platelet aggregation. Conversely, low-molecular-weight fucoidan can inhibit GPIb–vWF-mediated platelet aggregation, spreading, and thrombus formation [100]. Regarding procoagulant effects, C-type lectin-like receptor 2 (CLEC-2) serves as a physiological target through which fucoidan regulates platelet function in mice. Fucoidan activates mouse platelets via Src-, Syk-, and PLCγ2-dependent signaling pathways. In humans, fucoidan activates platelets through Src-, phosphoinositide 3-kinase (PI3K)-, and Akt-dependent but Syk-independent signaling cascades downstream of platelet endothelial aggregation receptor 1 (PEAR1). Meanwhile, GPIbα participates in both procoagulant and anticoagulant regulatory processes [101,102]. Regarding anticoagulant effects, sulfated fucoidan extracted from *Laminaria japonica* exerts anticoagulant activity by reducing thrombin activity following oral administration. It also exhibits significant antiplatelet effects by regulating the levels of platelet aggregation-related factors, including thromboxane B_2_ (TXB_2_) and 6-keto-PGF1α, and demonstrates favorable fibrinolytic activity [103].

It should be noted that the effects of fucoidan on the coagulation system are not uniformly anticoagulant. Although most studies support its anticoagulant and antithrombotic activities, these effects may be influenced by factors such as molecular weight, sulfation characteristics, purity, dose, and route of administration. Under certain conditions, fucoidan may exert different effects on the coagulation process. Therefore, fucoidan should be regarded as a structure-dependent modulator of coagulation homeostasis. Further studies are needed to define the safe dose range and potential bleeding risk in the context of cerebrovascular disease.

### 3.5. Gut Microenvironment

Fucoidan has demonstrated potential therapeutic effects against cancer, inflammatory bowel disease, diabetes, and obesity by repairing intestinal mucosal damage and improving the intestinal microenvironment. Previous studies have shown that, in non-obese diabetic mice, fucoidan improves intestinal permeability by reducing the levels of diamine oxidase (DAO), D-lactic acid (D-LA), fatty acid-binding protein 2 (FABP2), and endotoxin. It also enhances intestinal mucosal immune function by modulating Th1/Th2/Th17 responses. Furthermore, fucoidan improves intestinal barrier integrity by increasing the expression of tight junction proteins, including ZO-1, Claudin-1, and Occludin, thereby protecting intestinal mucosal function [104]. Similarly, in a DSS-induced ulcerative colitis model, fucoidan improves intestinal barrier function by regulating the expression of ZO-1, Claudin-2, and Occludin proteins. Meanwhile, it increases the abundance of Bacteroidetes and decreases the abundance of Fusobacterium, thereby alleviating intestinal inflammatory damage [68].

Fucoidan may also exert neuroprotective effects by improving the gut microenvironment. For example, in a chemotherapy-induced peripheral neuropathy model, fucoidan activates the p62/Keap1/Nrf2 signaling pathway, thereby enhancing the transcriptional expression of SR-A1. This effect improves the phagocytic capacity of macrophages and facilitates the clearance of HMGB1, LPS, and NETs. Meanwhile, fucoidan maintains gut microbiota diversity and composition, particularly by increasing the abundance of *Bacteroidetes* and *Verrucomicrobia*. Through the gut–blood–brainstem axis, fucoidan ultimately alleviates pathological damage associated with peripheral demyelination [105]. Furthermore, in a D-galactose-induced cognitive decline model, the neuroprotective effects of fucoidan may be associated with the maintenance of gut microbiota homeostasis. Fucoidan increases the abundance of *Bacteroidota*, *Muribaculaceae*, and *Akkermansia* while reducing *Firmicutes* abundance, thereby alleviating D-galactose-induced cognitive impairment [87]. However, direct evidence linking the gut–brain axis to VCI remains limited. In particular, the complete causal chain linking fucoidan, gut microbiota, cerebral microvasculature, and cognitive function has yet to be rigorously established. Therefore, the gut–brain axis may represent an important potential mechanism through which fucoidan exerts effects on VCI. At present, however, it is more appropriately considered a promising research direction rather than an established mechanism.

**Figure 2:**
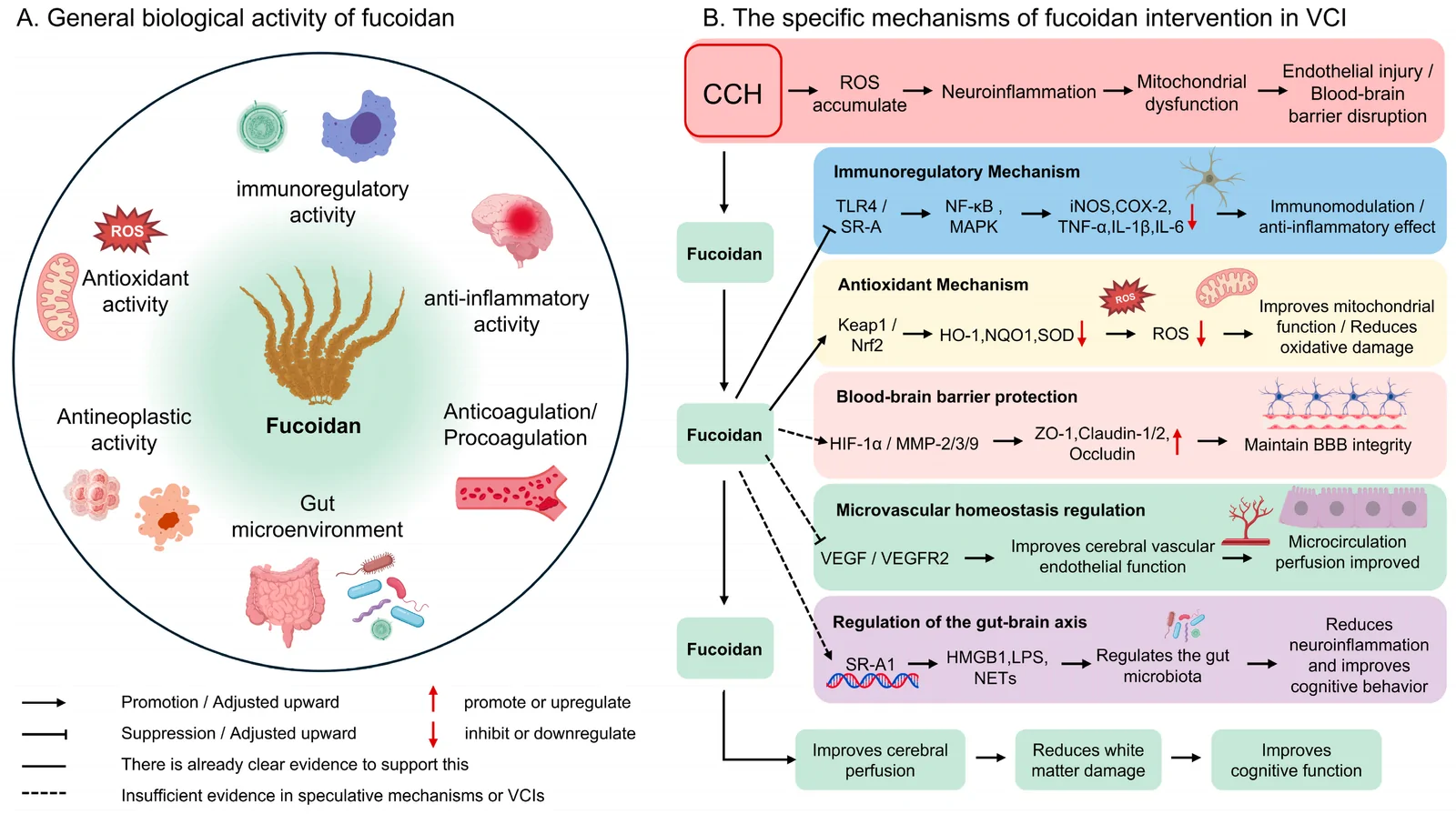
General biological activities of fucoidan and its potential mechanisms of action in vascular cognitive impairment. (**A**) Fucoidan exhibits a range of biological activities, including antioxidant, immunomodulatory, anti-inflammatory, anticoagulant/antithrombotic, and gut microenvironment-modulating effects. (**B**) In vascular cognitive impairment (VCI), chronic cerebral hypoperfusion (CCH) can induce the accumulation of ROS, neuroinflammation, mitochondrial dysfunction, and vascular endothelial/BBB impairment, thereby contributing to reduced cerebral perfusion, white matter injury, and cognitive decline. Fucoidan may improve cerebral perfusion, alleviate white matter injury, and delay VCI progression by modulating inflammation, oxidative stress, BBB integrity, microcirculatory homeostasis, and gut–brain axis-related pathways. Arrows indicate promotion or upregulation, whereas T-bars indicate inhibition. Solid lines represent mechanisms supported by existing evidence, whereas dashed lines indicate putative mechanisms or mechanisms for which direct evidence in VCI remains limited (created with BioGDP.com [106]).

## 4. The Possible Mechanism of Action of Fucoidan in Vascular Cognitive Impairment

Vascular cognitive impairment (VCI) is primarily driven by chronic cerebral hypoperfusion (CCH) [107]. Persistent hypoperfusion initiates a pathological cascade characterized by energy metabolism dysfunction, oxidative stress, and neuroinflammation. This cascade activates multiple molecular pathways involved in inflammatory responses and cellular injury, ultimately resulting in blood–brain barrier disruption, neuronal loss, and white matter lesions. Given the multifactorial nature of VCI, therapeutic strategies targeting multiple pathological processes simultaneously are considered particularly promising. Fucoidan, a sulfated marine polysaccharide with well-documented antioxidant, anti-inflammatory, endothelial-protective, and immunomodulatory properties, has therefore attracted increasing attention as a potential therapeutic agent for VCI.

Considerable heterogeneity exists among available studies with respect to algal source, molecular weight, degree of sulfation, extraction procedures, and administration regimens, which complicates direct comparisons across studies and warrants cautious interpretation of the current findings. Nevertheless, recent in vivo studies have provided important VCI-related, supportive evidence for the therapeutic potential of fucoidan against VCI-associated pathological changes, particularly cognitive impairment and vascular injury.

For example, a recent study evaluated the neuroprotective effects of fucoidan extracted from *Fucus vesiculosus*. In a non-VCI model of cognitive decline, oral administration (p.o.) of this fucoidan at a dose of 100 mg/kg/day significantly alleviated recognition memory impairment. The neuroprotective effect was attributed to modulation of the gut–brain axis through alterations in gut microbiota composition and systemic metabolism [108]. Furthermore, Cheng et al. demonstrated the vascular protective effects of fucoidan in an apolipoprotein E-deficient (*ApoE-*/*-*) mouse model of atherosclerosis, a major risk factor for VCI. Intraperitoneal injection of fucoidan at 60 mg/kg/day significantly reduced lipid accumulation and delayed carotid plaque progression by enhancing p62/SQSTM1-dependent selective autophagy and subsequently suppressing NLRP3 inflammasome activation [109]. Together, these studies—conducted in a model of cognitive decline and in a VCI-related vascular risk model (atherosclerosis) rather than in classical VCI or CCH models—provide indirect, supportive VCI-related evidence that fucoidan may exert multi-target neurovascular protection through mechanisms ranging from gut–brain axis regulation to selective autophagy enhancement and NLRP3 inflammasome inhibition, thereby mitigating cognitive dysfunction and vascular injury relevant to VCI pathogenesis.

Despite these encouraging findings, direct evidence supporting the therapeutic efficacy of fucoidan in classical VCI or chronic cerebral hypoperfusion (CCH) models remains limited. Most mechanistic insights into fucoidan are derived from non-VCI disease models, including cancer, metabolic disorders, and inflammatory diseases. Therefore, these findings should be regarded as indirect supportive evidence rather than definitive evidence for VCI. Nevertheless, many of the molecular pathways regulated by fucoidan are closely associated with interruption of the pathological cascade underlying VCI. The following sections discuss these mechanisms in detail, including the regulation of oxidative stress, neuroinflammation, apoptosis, blood–brain barrier integrity, and other key pathological processes.

### 4.1. CCH–VCI: Temporal Pathological Cascade and Multisite Intervention by Fucoidan

The potential effects of fucoidan on VCI can be integrated into a temporal four-stage pathological cascade model (Figure 3). Chronic cerebral hypoperfusion (CCH) initially induces mitochondrial dysfunction and excessive production of ROS, which may activate the MAPK/NF-κB signaling axis and amplify neuroinflammation. Subsequent activation of HIF-1α/MMPs may compromise BBB integrity, impair vascular endothelial function, and contribute to cerebral microcirculatory dysfunction. These pathological changes may ultimately lead to neuronal apoptosis, white matter injury, and cognitive decline. The potential value of fucoidan lies in its ability to act on multiple targets and pathological nodes. At the upstream level, fucoidan may attenuate oxidative stress through the Nrf2/HO-1 and PGC-1α pathways. It may also suppress neuroinflammation through the NF-κB/MAPK and NLRP3 pathways. In addition, modulation of PI3K/AKT, HIF-1α/MMPs, and VEGF-related signaling may contribute to the preservation of vascular endothelial and BBB integrity and the maintenance of cerebral microcirculation. Fucoidan may further exert neuroprotective effects through PI3K/AKT, BDNF, and anti-apoptotic mechanisms. Collectively, these effects may act at multiple points within the pathological cascade and potentially attenuate the interactions among oxidative stress, neuroinflammation, vascular dysfunction, and neuronal injury. This multitarget profile may be relevant to the complex and interconnected pathological processes underlying VCI. Regarding the timing of intervention, fucoidan may have greater potential during the early stages of VCI, when some pathological changes may remain modifiable. This stage may therefore represent a potentially relevant window for early intervention and secondary prevention in high-risk populations. During disease progression, continued protection of vascular endothelial function and cerebral microcirculation may help delay pathological deterioration. In the late stage, when substantial neuronal apoptosis and irreversible white matter injury have occurred, the potential for functional recovery may be limited. Under such circumstances, fucoidan may be more appropriately considered as an adjunctive component of combination therapy, with an emphasis on slowing disease progression and maintaining neurological function. Overall, the available evidence supports a potential role for fucoidan as an early intervention and adjunctive strategy throughout the disease course, although this temporal therapeutic framework requires further validation in appropriate VCI and CCH models.

### 4.2. Inhibiting the Core Pathways of Neuroinflammation in VCI

During the initiation and progression of VCI, CCH induces oxidative stress and excessive accumulation of ROS. Elevated ROS levels further activate the MAPK signaling pathway, including ERK, JNK, and p38 MAPK, resulting in abnormal glial activation and promoting NF-κB activation and nuclear translocation. Consequently, the sustained expression of pro-inflammatory mediators, such as TNF-α, IL-1β, and IL-6, is induced [96,110]. Meanwhile, reciprocal regulatory interactions exist between MAPK and NF-κB signaling, and these pathways jointly promote NLRP3 inflammasome activation, thereby amplifying neuroinflammatory responses. In particular, p38 MAPK also contributes to blood–brain barrier disruption and neuronal apoptosis, further aggravating neurofunctional impairment associated with VCI. Fucoidan attenuates these pathological processes by modulating the NF-κB/MAPK inflammatory signaling axis. On the one hand, fucoidan inhibits excessive NF-κB activation and reduces the production of pro-inflammatory mediators. Meanwhile, it may moderately regulate immune cell activity, including macrophages [67,111] and natural killer (NK) cells, thereby preventing excessive inflammatory responses while maintaining normal immune function. Consequently, fucoidan alleviates neurovascular unit damage caused by pathological inflammation [112,113].

On the other hand, fucoidan suppresses the phosphorylation of ERK, JNK, p38 MAPK, and other components of the MAPK signaling pathway. This regulation reduces inflammatory signaling activation and glial inflammatory responses, limits the amplification of inflammatory cascades, and improves the neuroinflammatory microenvironment under VCI conditions [114,115].

Furthermore, pro-inflammatory mediators contribute to blood–brain barrier disruption by activating microglia and inducing HIF-1α expression, which subsequently promotes MMP-2/3/9 activation [116]. Fucoidan exerts additional anti-inflammatory effects through regulation of downstream PI3K/AKT and Ras/MAPK signaling pathways, thereby suppressing HIF-1α activation and reducing MMP-2/3/9 activity.

It should be noted that the mechanisms described below remain largely hypothetical and are inferred from existing evidence. Direct experimental validation of the complete fucoidan–cGAS–STING–VCI pathway is currently lacking. Existing studies suggest that the cGAS–STING pathway may represent an important inflammatory regulatory node in the development and progression of VCI. Activation of this pathway may contribute to cognitive decline by mediating the sensing of DNA damage and promoting neuroinflammation, blood–brain barrier disruption, and white matter injury. Although direct experimental evidence linking the cGAS–STING pathway to VCI remains limited, the established antioxidant, mitochondrial protective, anti-inflammatory, blood–brain barrier-protective, and microglial regulatory effects of fucoidan are highly associated with the biological processes regulated by cGAS–STING signaling. We hypothesize that fucoidan may ameliorate vascular cognitive impairment by reducing oxidative stress-induced mitochondrial DNA (mtDNA) release and modulating cGAS–STING/NF-κB-mediated neuroinflammatory responses [43,117].

### 4.3. Interrupting the Oxidative–Inflammatory Cycle

In VCI, abnormalities in the electron transport chain (ETC) and mitochondrial dynamics contribute to cerebrovascular damage and increased ROS production [118]. ROS not only induce oxidative damage to lipids, proteins, and nucleic acids, but also serve as critical activators of inflammasomes, thereby establishing a vicious cycle of “oxidative stress–inflammatory activation”. Meanwhile, excessive ROS directly induce neuronal oxidative injury and downregulate tight junction proteins within the BBB, resulting in increased barrier permeability [119]. Fucoidan promotes the nuclear translocation of Nrf2 by activating the Nrf2/HO-1 signaling pathway, thereby upregulating the expression of antioxidant enzymes, including HO-1, SOD, and CAT. This process facilitates ROS scavenging during VCI progression and inhibits signal transduction through the Nox2–ROS–inflammatory pathway [120,121]. Furthermore, hippocampal PGC-1α expression is reduced in VCI, whereas fucoidan restores PGC-1α expression. This regulation improves neuronal energy metabolism under hypoxic conditions and enhances the expression of mitochondrial antioxidants and uncoupling proteins (UCPs), thereby reducing ROS generation. Meanwhile, fucoidan alleviates oxidative stress-induced damage to mitochondria, neurons, and the BBB; disrupts the vicious cycle between oxidative stress and inflammation; and suppresses the upstream pathological cascade involved in VCI progression [118].

### 4.4. Neuronal Protection

Several pathological pathways in VCI contribute to neuronal apoptosis and injury, including oxidative stress and NF-κB-mediated inflammatory signaling, impaired neuronal survival signaling caused by downregulation of BDNF, and inflammation and apoptosis aggravated by HIF-1α activation [122]. Meanwhile, CCH promotes Aβ deposition and, together with tau protein hyperphosphorylation, further exacerbates cognitive impairment. In addition, impaired oligodendrocyte maturation resulting from white matter lesions further contributes to neurological dysfunction [123].

Fucoidan exerts neuroprotective effects through multiple mechanisms and effectively alleviates neuronal damage associated with VCI. By modulating the PI3K/AKT signaling pathway, fucoidan may enhance neuronal survival, reduce apoptosis, and protect cerebral vascular function [124]. It directly counteracts oxidative stress- and inflammation-mediated apoptotic signaling pathways, including Bax/Bcl-2 and caspase-3 pathways, thereby reducing neuronal loss in key cognitive regions such as the hippocampus and frontal cortex [124,125]. Furthermore, fucoidan improves cognitive function and memory impairment by promoting neuronal dendritic growth and increasing dendritic spine density through BDNF upregulation [126]. Fucoidan can also inhibit HIF-1α activation, reduce matrix metalloproteinase (MMP)-2/3/9 activity, and attenuate cellular damage, thereby providing neuroprotective effects.

### 4.5. Regulating Vascular-Related Pathways

During the onset and progression of VCI, VEGF plays a critical and complex dual role in angiogenesis, endothelial function maintenance, and regulation of neurovascular unit homeostasis. Under conditions of chronic cerebral hypoperfusion, VEGF expression may be compensatorily upregulated, which contributes to vascular repair and collateral vessel formation during the early stages. However, persistent and excessive activation of VEGF signaling may induce abnormal angiogenesis, disrupt endothelial barrier integrity, and increase blood–brain barrier permeability, thereby exacerbating structural and functional abnormalities in the cerebral microvasculature [127]. Therefore, VEGF regulation should not be simply interpreted as a “higher is better” or “lower is better” phenomenon; rather, maintaining a moderate, dynamic, and coordinated signaling balance is essential. It should be noted that current evidence regarding the regulation of VEGF-related pathways by fucoidan is derived predominantly from non-VCI models, including studies of tumor angiogenesis, wound healing, and vascular endothelial function. Direct experimental evidence in the context of CCH or VCI remains limited. Previous non-VCI studies have demonstrated that fucoidan exhibits bidirectional and context-dependent regulation of VEGF-related pathways, with effects closely associated with its molecular weight. HMWF generally exhibits anti-angiogenic effects accompanied by reduced VEGF expression, whereas LMWF may promote angiogenesis and enhance VEGF-associated signaling activity [128,129,130]. Further studies have revealed that fucoidan activates VEGF signaling by enhancing the interaction between VEGF-165 and VEGFR-2/NRP1, thereby increasing microvascular density and improving endothelial function [131]. Meanwhile, fucoidan may promote mitochondrial protein kinase phosphorylation and activation of the PI3K/AKT/eNOS signaling pathway, leading to increased VEGF expression and improved endothelial-dependent vasodilation [132]. Conversely, as discussed above, fucoidan may suppress excessive VEGF activation through modulation of the PI3K/AKT, Ras/MAPK, and other signaling pathways. These findings suggest that fucoidan does not simply exert unidirectional stimulation or inhibition of VEGF signaling. Instead, it may adaptively regulate VEGF activity to maintain signaling homeostasis under different pathological stages and tissue microenvironments.

However, these findings primarily reflect the potential of fucoidan to modulate the VEGF pathway under different experimental conditions and cannot yet be directly interpreted as evidence of its definitive effects on cerebrovascular homeostasis in the specific pathological context of VCI. Based on this concept, we hypothesize that, in the context of chronic cerebral hypoperfusion-induced VCI, fucoidan may restore the dynamic balance of VEGF expression and downstream signaling pathways, thereby re-establishing cerebral microvascular homeostasis, improving endothelial function, and enhancing microcirculatory perfusion. Consequently, fucoidan may alleviate neurovascular damage caused by chronic cerebral hypoperfusion and disrupt the vicious cycle of “insufficient cerebral perfusion–microvascular injury–further perfusion decline”. Furthermore, improved endothelial function may preserve blood–brain barrier integrity and reduce permeability, thereby suppressing subsequent inflammatory responses, oxidative stress, and neuronal damage, ultimately slowing the pathological progression of VCI [123,133,134]. Nevertheless, this hypothesis requires further validation through direct experimental studies using relevant VCI or CCH models.

### 4.6. Adjuvant Anticoagulation/Antithrombotic Therapy

Fucoidan may ameliorate coagulation disorders and cerebral microcirculatory dysfunction associated with VCI through multi-target regulation of platelet activation, the coagulation cascade, and fibrinolytic system homeostasis. Under the influence of aging, hypertension, and other risk factors, cerebral microvascular endothelial injury leads to exposure of the subendothelial matrix. This process triggers the release of vWF and promotes its interaction with platelet membrane glycoprotein GPIbα, thereby mediating platelet adhesion, activation, and subsequent thrombus formation [135]. Meanwhile, endothelial dysfunction can activate the tissue factor-dependent coagulation pathway, initiating sequential coagulation factor activation, accelerating thrombin generation and fibrin deposition, and consequently exacerbating cerebral microvascular stenosis and hypoperfusion [116]. Fucoidan, particularly low-molecular-weight fucoidan, can competitively inhibit vWF–GPIbα-mediated platelet adhesion by binding to the extracellular domain of GPIbα, thereby suppressing abnormal platelet aggregation. In addition, fucoidan activates heparin cofactor II (HCII) and inhibits the activities of coagulation factor Xa and thrombin, thereby interfering with the intrinsic coagulation cascade, reducing thrombin generation and fibrin deposition, and ultimately preventing intravascular thrombus formation and progression of vascular stenosis [136]. Furthermore, fucoidan enhances fibrinolytic activity, promotes fibrin degradation and thrombus clearance, and maintains platelet–endothelial homeostasis by reducing TXB_2_ levels and increasing 6-keto-PGF1α levels, which exert antiplatelet effects. These actions collectively contribute to improved cerebral microcirculatory perfusion [103]. In summary, fucoidan exerts synergistic protective effects against coagulation dysfunction associated with VCI through multiple mechanisms. It inhibits platelet adhesion, regulates the coagulation cascade, promotes fibrinolysis, and maintains platelet–vascular endothelial homeostasis. Consequently, fucoidan reduces microvascular thrombus formation, improves cerebral tissue perfusion, and may delay the onset and progression of VCI.

### 4.7. Blood–Brain Barrier Protection

BBB disruption is an important event in the progression of VCI. Current evidence suggests that fucoidan may indirectly protect the BBB by exerting anti-inflammatory and antioxidant effects and improving endothelial function. These effects may involve the regulation of pathways such as NF-κB/MAPK and HIF-1α/MMPs, thereby helping maintain the expression of tight junction proteins and preserving BBB integrity [137]. However, direct evidence supporting a BBB-protective effect of fucoidan in VCI remains limited. Meanwhile, the oral absorption and brain availability of fucoidan may be limited. Meanwhile, fucoidan is limited by low bioavailability and poor BBB permeability [138]. Therefore, the protective effects of fucoidan on VCI may involve a combination of peripheral regulation and indirect central effects. Further pharmacokinetic, tracing, and pharmacodynamic studies are required to clarify these relationships.

## 5. Challenges and Prospects

### 5.1. Challenges in Clinical Translation

From the perspective of clinical translation, fucoidan still faces several challenges. First, current human studies remain largely at an early stage. Most have involved small sample sizes and short-term oral interventions. Research has mainly focused on gastrointestinal regulation, metabolic improvement, and nutritional supplementation. Large-scale, multicenter clinical studies specifically addressing VCI and other cerebrovascular cognitive disorders remain lacking. Second, the pharmacokinetic characteristics of fucoidan have not been fully elucidated. In particular, its absorption, distribution, metabolism, and excretion in humans remain insufficiently characterized. This uncertainty may limit the optimization of dosage and the design of appropriate administration regimens. Third, fucoidan is a structurally heterogeneous marine polysaccharide. Its biological activity can be influenced by multiple factors, including algal source, extraction and purification procedures, molecular weight distribution, and sulfation patterns. These factors present considerable challenges for batch-to-batch consistency and the establishment of standardized quality-control criteria. In addition, the clinical translatability of preclinical evidence remains limited. In vitro studies can help elucidate the regulatory effects of fucoidan on inflammation, oxidative stress, endothelial function, and apoptosis. However, experimental conditions are generally simplified and cannot fully recapitulate the complex pathological environment of VCI, which involves chronic cerebral hypoperfusion, BBB disruption, interactions among glial cells, and cerebral microvascular remodeling. Animal studies provide evidence that more closely reflects integrated pathological processes but are still subject to limitations, including the use of relatively limited disease models, interspecies differences, and discrepancies between experimental administration protocols and clinical practice. Therefore, neither in vitro nor in vivo studies alone currently provide high-level clinical evidence for the efficacy of fucoidan in VCI. The available findings should be interpreted in conjunction with pharmacokinetic data, safety assessments, and clinical evidence.

### 5.2. Administration Routes and Application Limitations

Regarding the route of administration, oral delivery of fucoidan is convenient and has been generally well tolerated in short-term studies, and it may be suitable for long-term intervention, although its long-term safety has not yet been established. However, its absorption in the gastrointestinal tract may be limited by its high molecular weight, resulting in relatively low systemic bioavailability. Oral administration may therefore be more suitable for intestinal effects, immunometabolism regulation, and interventions involving the gut–brain axis. In contrast, intravenous administration can substantially increase systemic exposure and may enhance systemic anti-inflammatory and vascular-protective effects. However, it may also increase exposure to the anticoagulant effects of fucoidan, potentially increasing the risk of bleeding. Current evidence for intravenous administration is derived primarily from animal studies, and human pharmacokinetic and safety data remain limited. Therefore, the clinical potential of intravenous fucoidan administration requires further investigation.

### 5.3. Dose–Response Relationships and Safety Considerations

It is noteworthy that studies investigating the dose–response relationship of fucoidan remain limited, and the comparability of findings across studies is constrained. This is largely attributable to substantial differences in algal source, molecular weight, degree of sulfation, extraction and purification procedures, and route of administration. Available evidence suggests that low-molecular-weight fucoidan may exhibit favorable activity and bioavailability in terms of anti-inflammatory, antioxidant, and antithrombotic effects. However, these effects do not necessarily follow a simple linear dose–response relationship, and some biological effects may exhibit threshold or plateau effects. In the context of VCI, defining the dose–response relationship is further complicated by the complexity of disease progression, limited oral absorption of fucoidan, and potentially insufficient central exposure. Some toxicological studies reported that no obvious signs of toxicity or death were observed in animals and humans after taking fucoidan. Sargassum-derived brown algal polysaccharide did not cause animal death, obvious toxicity, or significant abnormalities in indicators such as body weight, organ weight, food intake and water consumption in the acute toxicity test (single oral administration at 2000 mg/kg for 14 days) and subacute toxicity test (oral administration at 100–400 mg/kg for 28 days), exhibiting favorable safety [139]. Studies on oral fucoidan administration in humans mainly demonstrate good short-term tolerability. Twenty-three healthy volunteers took 4.05 g of *mozuku* fucoidan (derived from *Cladosiphon okamuranus*) daily. After two weeks, only total cholesterol and LDL decreased, while liver and kidney indicators remained unchanged [140]. No clinically significant changes in safety indicators or drug-related adverse events were observed in patients with osteoarthritis after 12-week treatment with 300 mg of brown algae fucoidan per day [141]. Overall, because the available human studies were predominantly short-term, these findings support the short-term tolerability of fucoidan rather than its long-term safety, which has not yet been established; additional long-term clinical studies are therefore still needed to further characterize the pharmacokinetics and long-term safety profile of fucoidan in humans [142]. Therefore, systematic studies using standardized fucoidan preparations are needed to evaluate dose gradients, pharmacokinetic–pharmacodynamic relationships, and safety profiles. Such studies may help define the effective dose range, optimal therapeutic window, and safety margins of fucoidan.

### 5.4. Optimization Strategies and Technological Approaches

To address the challenges described above, future research should focus on developing practical strategies to facilitate the clinical translation of fucoidan. First, greater attention should be paid to the standardized preparation and detailed structural characterization of fucoidan. Traceable sources, consistent processing procedures, and robust quality-control systems should be established to improve batch-to-batch consistency and further clarify structure–activity relationships. Second, the preparation of low-molecular-weight fucoidan and chemical or enzymatic modification may provide approaches for retaining its anti-inflammatory and vascular-protective activities while potentially reducing anticoagulant-related risks. However, the effects of such modifications on biological activity and safety require systematic evaluation. Third, emerging formulation and delivery strategies, including nanoparticles, liposomes, hydrogels, and colon-targeted delivery systems, may improve the stability, absorption, and tissue distribution of fucoidan. These approaches may therefore provide potential strategies for enhancing bioavailability while reducing systemic adverse effects. For VCI, the integration of cerebrovascular- or endothelial-targeted delivery systems may further improve the delivery of fucoidan to relevant vascular compartments and potentially enhance its neurovascular protective effects. Further preclinical and pharmacokinetic studies are required to determine the feasibility and safety of these approaches.

### 5.5. Potential Clinical Positioning of Fucoidan in VCI

In addition, the potential role of fucoidan in VCI should be considered within the broader context of candidate neuroprotective interventions. Compared with single-target agents, fucoidan may offer a potential advantage through its ability to modulate multiple interconnected pathological processes, including inflammation, oxidative stress, endothelial dysfunction, coagulation imbalance, and BBB disruption. This multitarget activity may be relevant to the complex and multifactorial pathophysiology of VCI. However, several limitations should also be considered, including its high structural heterogeneity, incompletely characterized pharmacokinetic properties, uncertain central nervous system exposure, and challenges in standardization. Therefore, at the current stage of evidence, fucoidan may be more appropriately considered as a candidate for adjunctive or preventive intervention with potential multitarget regulatory effects, rather than as an established therapeutic agent intended to replace current standard treatments for VCI.

### 5.6. Conclusions and Future Perspectives

This review adopts a cautious approach to evaluating the consistency and strength of the available evidence. The bidirectional effects of fucoidan on processes such as angiogenesis and coagulation should not necessarily be regarded as contradictory. Rather, they may reflect its high structural heterogeneity and the resulting structure- and context-dependent biological activities. The pro- and anti-angiogenic effects of fucoidan have been reported in different experimental settings, including tumor models and models of ischemia or wound healing. Similarly, its effects on coagulation may vary with molecular weight and other structural characteristics, supporting its potential role as a structure-dependent modulator of coagulation homeostasis. However, most mechanistic evidence currently originates from non-VCI models or in vitro experiments and therefore provides only indirect or preliminary support. Such findings should not be directly extrapolated to patients with VCI. Several limitations remain to be addressed, including substantial differences in biological activity associated with algal sources and processing procedures, uncertainty regarding whether cognitive benefits observed in CCH models are primary or secondary effects, an incompletely defined therapeutic window, potentially nonlinear dose–response relationships, unclear safety margins for anticoagulant activity, and difficulties in standardizing structurally heterogeneous natural products. These limitations serve to define the current boundaries of the evidence rather than to exclude the potential of fucoidan as an intervention for VCI. Instead, they highlight the need for standardized fractions, rigorous mechanistic validation, and well-designed clinical studies.

Overall, fucoidan has a biologically plausible rationale and a preliminary research basis for potential applications in the prevention and management of VCI. However, its clinical translation remains at an exploratory stage. Future research should systematically address key issues, including standardized preparation, pharmacokinetic evaluation, short- and long-term safety assessment, optimization of delivery systems, and biomarker-guided clinical trial design. These efforts will be essential for determining the actual therapeutic potential and clinical applicability of fucoidan in VCI.

## Figures and Tables

**Figure 1:**
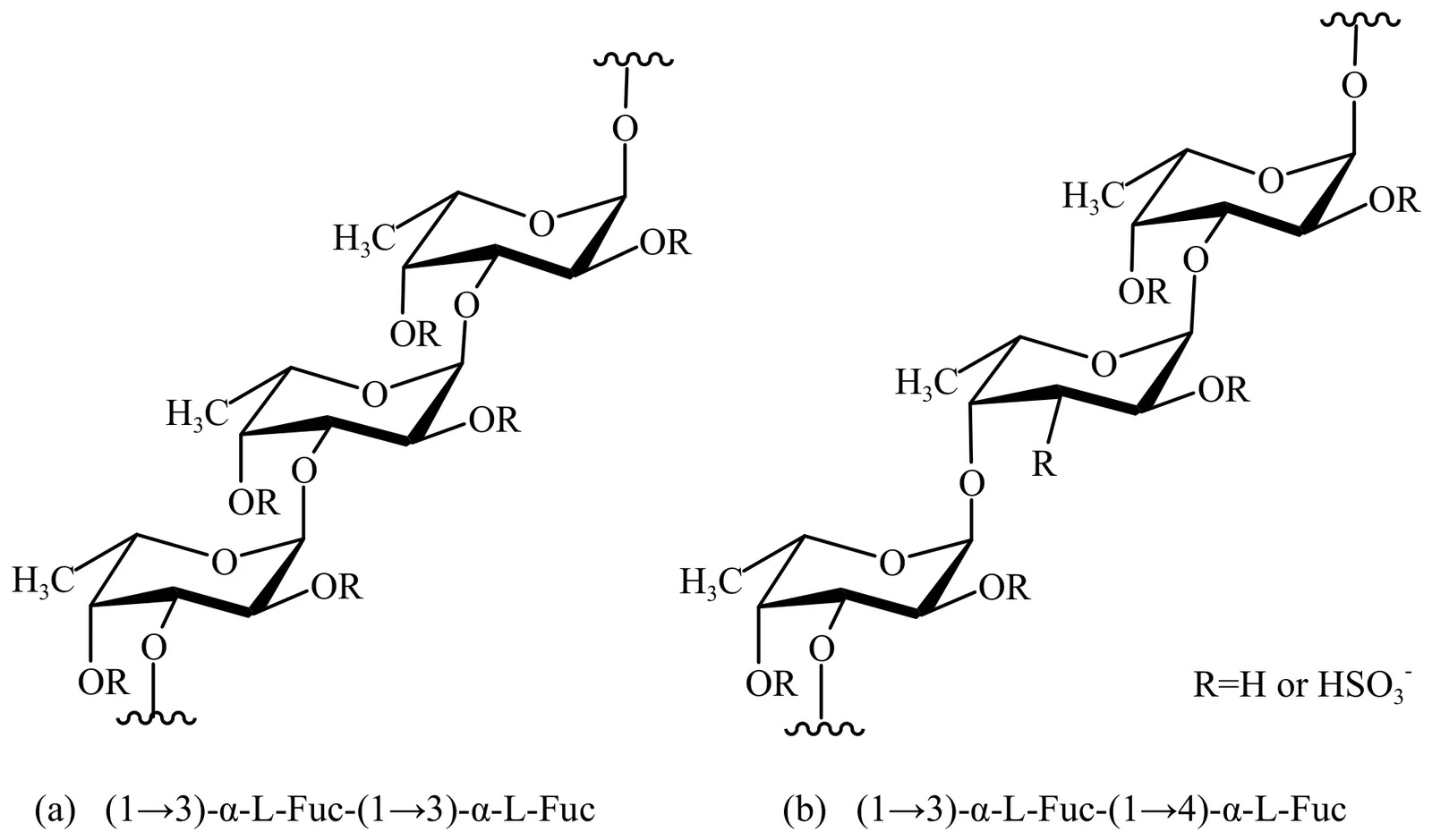
Chemical structure of fucoidan. L-fucose linked via an α-(1 → 3) bond (**a**) (Type I); L-fucose linked via alternating α-(1 → 3) and α-(1 → 4) bonds (**b**) (Type II). R = H or HSO_3_^−^.

**Figure 3:**
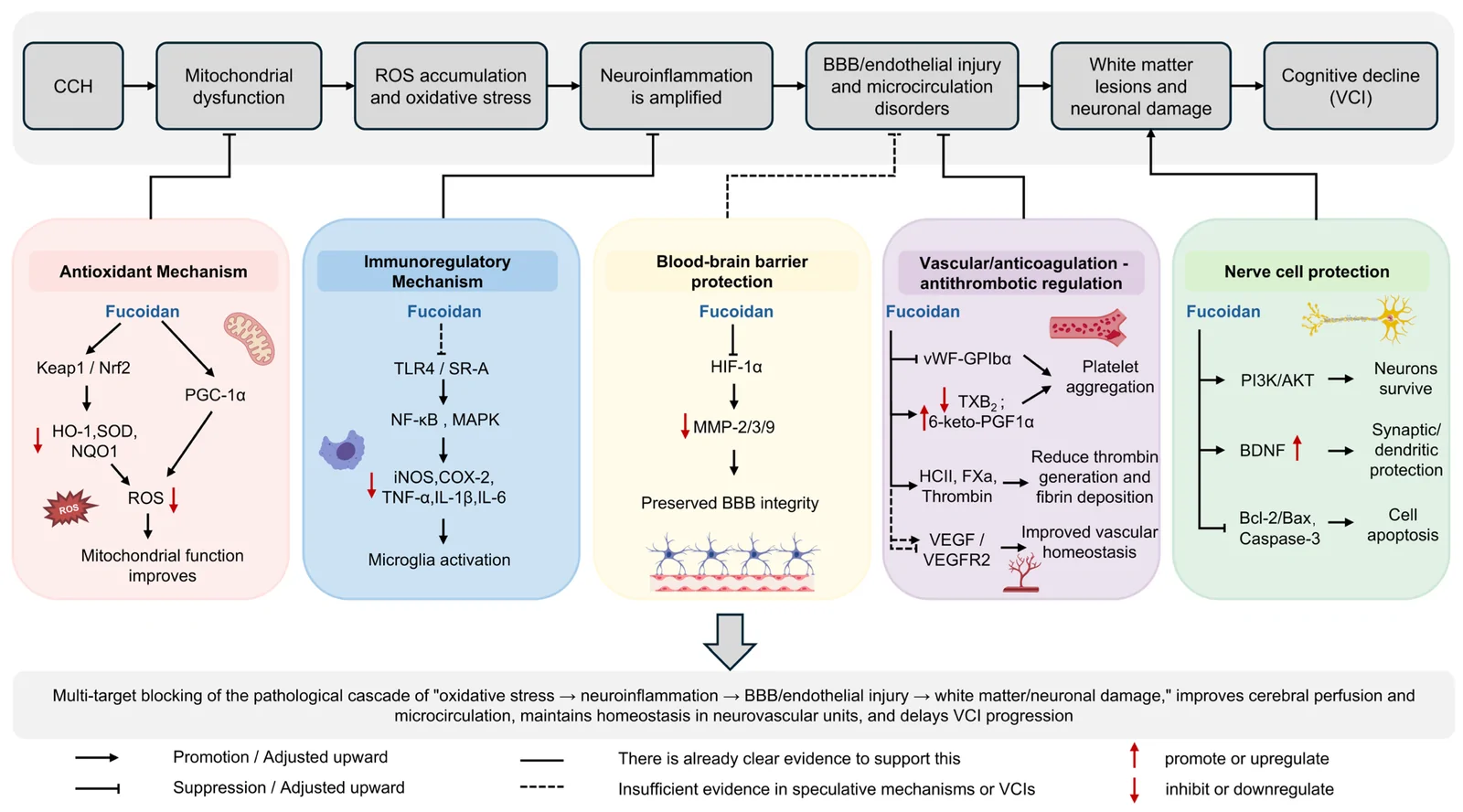
Schematic progression from chronic cerebral hypoperfusion to cognitive decline and potential intervention points of fucoidan. The flowchart illustrates the pathological cascade from chronic cerebral hypoperfusion (CCH) to vascular cognitive impairment (VCI), including mitochondrial dysfunction, oxidative stress, exacerbated neuroinflammation, BBB/endothelial injury accompanied by microcirculatory dysfunction, and subsequent white matter and neuronal injury. Fucoidan may intervene at multiple stages of this process through its antioxidant, anti-inflammatory, BBB-protective, vascular-regulatory, and neuroprotective effects, thereby potentially slowing the progression of cognitive decline. Solid lines indicate mechanisms supported by existing evidence, whereas dashed lines indicate putative mechanisms or mechanisms for which direct evidence in VCI remains limited (created with BioGDP.com [106]).

## Data Availability

The original contributions presented in this study are included in the article/Supplementary Material. Further inquiries can be directed to the corresponding authors.

## Supplementary Materials

The following supporting information can be downloaded at: https://www.mdpi.com/article/10.3390/md24100341/s1, Table S1: A Study on the Structural Characteristics and Biological Activity of Fucoidan; Table S2: Structural Characteristics, Preparation Techniques and Bioactivity of Low-Molecular-Weight Fucoidan.
