# Supplementary material for: Recent Advances in Fucoidan Bioactivity and Its Molecular Mechanisms in the Intervention of Vascular Cognitive Impairment
Source: Mar Drugs. 2026 Sep 28;24(10):341. doi: 10.3390/md24100341 (PMC13705966; doi:10.3390/md24100341)
Supplement: Supplementary file 1 [file marinedrugs-24-00341-s001.zip › marinedrugs-4524301-supplementary.pdf]

marine drugs

supplementary materials

To illustrate the structural characteristics and biological activities of fucoidan for the system, this supplementary material provides the following two summary tables. Table S1 summarizes the sources, molecular weights, preparation methods, structural characteristics, glycosidic-linkage patterns, and reported biological activities of fucoidan documented in literatures. Table S2 further focuses on low-molecular-weight fucoidan, summarizes its preparation methods, structural characteristics and biological activities, and compares the activity differences between low-molecular-weight and high-molecular-weight fucoidan.

Table S1: A Study on the Structural Characteristics and Biological Activity of Fucoidan

**Molecular weight（kDa）**	**Source**	**Preparation method**	**Structural features**	**Glycosidic linkage pattern**	**Reported bioactivity**
<10	Laminaria japonica (Rongcheng, Shandong, China)	Hot-water extraction followed by radical degradation	NR	Mainly (1→3)-linked α-L-fucopyranose residues, with a few (1→4)-linked α-L-fucopyranose residues	Activates the AMPK/mTOR-mediated autophagy pathway and exerts anti-inflammatory effects [1,2]
>300	Undaria pinnatifida (Shandong, China)	Extracted in water at 70°C overnight in the presence of $CaCl_2$	Monosaccharide composition: fucose, galactose, and glucose; sulfate content: 39.44%; uronic acid content: 4.27% [3,4]	(1→3)-D-galactose-(1→3)-L-fucose and (1→3)-D-galactose-(1→4)-L-fucose	NR
420	Ascophyllum nodosum / Lessonia trabeculata	Polysaccharides extracted using selective solvents (EtOH and Milli-Q H_2O) [5]	Fucose (52.1%), galactose (6.1%), glucose (21.3%), and xylose (16.5%) [6]	Repeating disaccharide unit: [→3)-α-L-Fuc($2SO_3^-$)-(1→4)-α-L-Fuc($2,3SO_3^-$)-(1→]	Possesses antitumor activity, promotes apoptosis, and inhibits proliferation [7,8]
469	Laminaria hyperborea	Mild acid degradation of pFuc with HCl at pH 3.0 and 70°C for 30 min	Composed of 97.8% fucose and 2.2% galactose	Predominantly (1→3)-α-L-fucopyranose, with 1→2- and 1→4-linked residues	Exhibits the most potent anticoagulant activity [9]
100-1600	Fucus serratus L.	NR	Predominantly L-fucosylated with some galactose incorpo-	→3)-α-L-Fucp-(1→4)-α-L-Fucp-(1→	NR

		rated (alternating polymer) [10]	

Abbreviations: NR, not reported.
Note:"NR" indicates that the corresponding information was not provided in the original reference rather than omitted by the authors. Due to differences in source species, extraction/purification procedures, and structural characterization methods, the reported structural and bioactivity data may not be directly comparable across studies.

Table S2: Structural Characteristics, Preparation Techniques and Bioactivity of Low-Molecular-Weight Fucoidan

**Molecular weight（kDa）**	**Preparation method**	**Structure features**	**Reported biological activity**
<10, ≤38	γ-ray irradiation	NR	Enhanced anticancer activity and significantly improved antiproliferative effects [11-13]
63.9±2.40	Acid hydrolysis	NR	Enhances immunoregulatory activity [14]
<6	Enzymatic hydrolysis using endo-α-1,4-L-fucoidanase	endo-α-1,4-L-fucoidanase	Antidiabetic activity; depolymerization products also exhibit antibacterial activity [15,16]
90	Photocatalytic degradation	Tetrasaccharide containing three sulfate groups and three fucose residues	Selectively inhibits the blood coagulation cascade; fucoidan with a molecular weight of 10–300 kDa exhibits the strongest anticoagulant activity [17,18]
20-30	Ultrasound-assisted degradation	NR	Greater anticancer activity [19]
2.6	Hydrogen peroxide degradation	α-(1→3)-L-fucose backbone, with sulfation occurring mainly at the C2 or C4 position	Anti-obesity activity; higher secondary antioxidant activity; enhanced anti-inflammatory activity [20-22]
<6	Hydrothermal degradation	α-L-Fucp2S-(1→3) and α-L-Fucp-(1→3) residues reported	Regulates alterations in the gut microbiota [23,24]

Abbreviations: NR, not reported.

Note: "NR" indicates that the corresponding structural information was not provided in the original reference rather than omitted by the authors. Due to differences in degradation method, structural characterization depth, and source material, direct comparison among studies should be interpreted with caution.

References

1. Chen, M.; Wang, J.; Zhang, P.; Jiang, Z.; Chen, S.; Liang, S.; Ma, T.; Liao, H.; Tan, W.; Niu, C.; et al. Low molecular weight fucoidan induces M2 macrophage polarization to attenuate inflammation through activation of the AMPK/mTOR autophagy pathway. *Eur J Pharmacol* **2025**, *986*, 177134, doi:10.1016/j.ejphar.2024.177134.
2. Chen, A.; Lan, Y.; Liu, J.; Zhang, F.; Zhang, L.; Li, B.; Zhao, X. The structure property and endothelial protective activity of fucoidan from Laminaria japonica. *Int J Biol Macromol* **2017**, *105*, 1421–1429, doi:10.1016/j.ijbiomac.2017.07.148.
3. Yang, Y.; Wang, N.; Hua, Y.; Wei, B.; Wang, H.; Yu, Y. Developments in the analytical methodologies and structure-activity relationships of low molecular weight fucoidans: A review. *Int J Biol Macromol* **2025**, *328*, 147544, doi:10.1016/j.ijbiomac.2025.147544.
4. Yu, J.; Li, Q.; Wu, J.; Yang, X.; Yang, S.; Zhu, W.; Liu, Y.; Tang, W.; Nie, S.; Hassouna, A.; et al. Fucoidan Extracted From Sporophyll of Undaria pinnatifida Grown in Weihai, China - Chemical Composition and Comparison of Antioxidant Activity of Different Molecular Weight Fractions. *Front Nutr* **2021**, *8*, 636930, doi:10.3389/fnut.2021.636930.
5. Foley, S.A.; Szegezdi, E.; Mulloy, B.; Samali, A.; Tuohy, M.G. An unfractionated fucoidan from Ascophyllum nodosum: extraction, characterization, and apoptotic effects in vitro. *J Nat Prod* **2011**, *74*, 1851–1861, doi:10.1021/np200124m.
6. Li, X.; Yang, C.; Wang, L.; Ullah, I.; Liu, X.; Feng, C.; Liu, Q. Exploring the effect and mechanism of fucoidan on liver cancer depending on network pharmacology and experimental verification. *Front Chem* **2025**, *13*, 1638785, doi:10.3389/fchem.2025.1638785.
7. Rui, X.; Pan, H.F.; Shao, S.L.; Xu, X.M. Anti-tumor and anti-angiogenic effects of Fucoidan on prostate cancer: possible JAK-STAT3 pathway. *BMC Complement Altern Med* **2017**, *17*, 378, doi:10.1186/s12906-017-1885-y.
8. Vishchuk, O.S.; Sun, H.; Wang, Z.; Ermakova, S.P.; Xiao, J.; Lu, T.; Xue, P.; Zvyagintseva, T.N.; Xiong, H.; Shao, C.; et al. PDZ-binding kinase/T-LAK cell-originated protein kinase is a target of the fucoidan from brown alga Fucus evanescens in the prevention of EGF-induced neoplastic cell transformation and colon cancer growth. *Oncotarget* **2016**, *7*, 18763–18773, doi:10.18632/oncotarget.7708.
9. Kopplin, G.; Rokstad, A.M.; Mélida, H.; Bulone, V.; Skjåk-Bræk, G.; Aachmann, F.L. Structural Characterization of Fucoidan from Laminaria hyperborea: Assessment of Coagulation and Inflammatory Properties and Their Structure-Function Relationship. *ACS Appl Bio Mater* **2018**, *1*, 1880–1892, doi:10.1021/acsabm.8b00436.
10. Bilan, M.I.; Grachev, A.A.; Shashkov, A.S.; Nifantiev, N.E.; Usov, A.I. Structure of a fucoidan from the brown seaweed Fucus serratus L. *Carbohydr Res* **2006**, *341*, 238–245, doi:10.1016/j.carres.2005.11.009.
11. Li, P.; Xiong, C.; Huang, W. Gamma-Irradiation-Induced Degradation of the Water-Soluble Polysaccharide from Auricularia polytricha and Its Anti-Hypercholesterolemic Activity. *Molecules* **2022**, *27*, 1110. doi:10.3390/molecules27031110.
12. Choi, J.I.; Kim, H.J. Preparation of low molecular weight fucoidan by gamma-irradiation and its anticancer activity. *Carbohydr Polym* **2013**, *97*, 358–362, doi:10.1016/j.carbpol.2013.05.002.
13. Yang, X.; Wang, S.; Trangle, S.S.; Li, Y.; White, W.L.; Li, J.; Ying, T.; Kong, Q.; Zhao, Y.; Lu, J. Investigation of Different Molecular Weight Fucoidan Fractions Derived from New Zealand Undaria pinnatifida in Combination with GroA Therapy in Prostate Cancer Cell Lines. *Mar Drugs* **2018**, *16*, 454. doi:10.3390/md16110454.
14. Borazjani, N.J.; Tabarsa, M.; You, S.; Rezaei, M. Improved immunomodulatory and antioxidant properties of unrefined fucoidans from Sargassum angustifolium by hydrolysis. *J Food Sci Technol* **2017**, *54*, 4016–4025, doi:10.1007/s13197-017-2867-2.
15. Zhao, Y.; Ning, L.; Zhu, P.; Jiang, J.; Yao, Z.; Zhu, B. The Origin, Properties, Structure, Catalytic Mechanism, and Applications of Fucoidan-Degrading Enzymes. *Mar Drugs* **2025**, *23*, 97. doi:10.3390/md23030097.
16. Qiu, Y.; Jiang, H.; Dong, Y.; Wang, Y.; Hamouda, H.I.; Balah, M.A.; Mao, X. Expression and Biochemical Characterization of a Novel Fucoidanase from Flavobacteriumalgicola with the Principal Product of Fucoidan-Derived Disaccharide. *Foods* **2022**, *11*, 1205. doi:10.3390/foods11071025.

17. Qi, Y.; Wang, L.; You, Y.; Sun, X.; Wen, C.; Fu, Y.; Song, S. Preparation of Low-Molecular-Weight Fucoidan with Anticoagulant Activity by Photocatalytic Degradation Method. *Foods* **2022**, *11*, 822. doi:10.3390/foods11060822.
18. Luthuli, S.; Wu, S.; Cheng, Y.; Zheng, X.; Wu, M.; Tong, H. Therapeutic Effects of Fucoidan: A Review on Recent Studies. *Mar Drugs* **2019**, *17*, 487.doi:10.3390/md17090487.
19. Ummat, V.; Sivagnanam, S.P.; Rai, D.K.; O'Donnell, C.; Conway, G.E.; Heffernan, S.M.; Fitzpatrick, S.; Lyons, H.; Curtin, J.; Tiwari, B.K. Conventional extraction of fucoidan from Irish brown seaweed Fucus vesiculosus followed by ultrasound-assisted depolymerization. *Sci Rep* **2024**, *14*, 6214, doi:10.1038/s41598-024-55225-z.
20. Chen, C.Y.; Wang, S.H.; Huang, C.Y.; Dong, C.D.; Huang, C.Y.; Chang, C.C.; Chang, J.S. Effect of molecular mass and sulfate content of fucoidan from Sargassum siliquosum on antioxidant, anti-lipogenesis, and anti-inflammatory activity. *J Biosci Bioeng* **2021**, *132*, 359–364, doi:10.1016/j.jbiosc.2021.06.005.
21. Yu, Y.; Zhao, Y.; Wang, H.; Hua, Y.; Yu, W.; Wang, N.; Wei, B.; Wang, H. A low molecular weight fucoidan with high sulfation has anti-obesity activity via gut microbiota regulation. *Carbohydr Polym* **2025**, *364*, 123739, doi:10.1016/j.carbpol.2025.123739.
22. Koh, H.S.A.; Lu, J.; Zhou, W. Structure characterization and antioxidant activity of fucoidan isolated from Undaria pinnatifida grown in New Zealand. *Carbohydr Polym* **2019**, *212*, 178–185, doi:10.1016/j.carbpol.2019.02.040.
23. Flórez-Fernández, N.; González-Muñoz, M.J.; Domínguez, H. Feasibility of posthydrolysis processing of hydrothermal extracts from Sargassum muticum. *Algal Research* **2017**, *27*, 73–81, doi:10.1016/j.algal.2017.08.002.
24. Shi, D.; Qi, J.; Zhang, H.; Yang, H.; Yang, Y.; Zhao, X. Comparison of hydrothermal depolymerization and oligosaccharide profile of fucoidan and fucosylated chondroitin sulfate from Holothuria floridana. *Int J Biol Macromol* **2019**, *132*, 738–747, doi:10.1016/j.ijbiomac.2019.03.127.
